# Risk factors for diabetic at-risk foot disease among the diabetes over 60 years old: a cross-sectional study

**DOI:** 10.3389/fmed.2026.1818395

**Published:** 2026-04-15

**Authors:** Man Wu, Song Xiao, Shuying Ye, Ping Huang, Dayin Zheng, Xiaoting Wang, Wenxuan Zou, Hupao Chen, Demei Chen, Xiaohong Li, Xueping Xu, Fengxiong Wang

**Affiliations:** 1 Department of Endocrinology, The Third Hospital of Xiamen, Xiamen, Fujian, China; 2 Department of Hand and Foot Surgery, The Third Hospital of Xiamen, Xiamen, Fujian, China; 3 Department of Ministry of Communities, The Third Hospital of Xiamen, Xiamen, Fujian, China

**Keywords:** at-risk foot, diabetic foot, glycemic control, peripheral neuropathy, peripheral vascular disease

## Abstract

**Background:**

Most studies related to at-risk foot are based on databases, cohorts, hospitalized patients, or outpatient studies, lacking comprehensive data on risk factors. According to the latest Diabetic foot risk stratification system, there is currently a lack of research or descriptions of the diabetic at-risk foot population.

**AIMs:**

This study aims to investigate the prevalence, characteristics, and influencing factors of at-risk foot conditions in the diabetic population over 60 years old.

**Methods:**

The study included 2003 diabetic patients from the Diabetic Foot Screening Project in Xiamen, China, from June 2022 to June 2023. Data were collected through questionnaires, physical examinations, and fasting blood glucose. All patients underwent screenings for three major categories: foot deformities, LOPS (Loss of Protective Sensation), and PAD (Peripheral Arterial Disease).

**Results:**

The prevalence of at-risk foot conditions among diabetics over 60 years old was 33.50%. After adjusting for potential confounders, each 1 mmol/L increase in fasting blood glucose (FBG) was associated with a 4.8% increase in the probability of at-risk foot condition (OR = 1.048, 95% CI: 1.018–1.078, *p* = 0.001). Additionally, for every 1 dollar increase in income, the probability of at-risk foot condition increased by 37.4% (OR = 1.374, 95% CI: 1.022–1.848, *p* = 0.035). In contrast, the presence of hypertension reduced the probability of at-risk foot condition by 37.4% (OR = 0.626, 95% CI: 0.464–0.845, *p* = 0.002). Cardiovascular disease (CVD) significantly increased the probability of at-risk foot condition by approximately 90.6% (OR = 1.906, 95% CI: 1.091–3.328, *p* = 0.023). Cerebrovascular disease (CBD) significantly reduced the probability (OR = 0.226, 95% CI: 0.064–0.803, *p* = 0.022), while kidney disease (KD) significantly increased it by approximately 257.8% (OR = 3.578, 95% CI: 1.567–8.171, *p* = 0.025). Notably, peripheral neuropathy (PN) significantly increased the probability of at-risk foot condition by approximately 882.9% (OR = 9.829, 95% CI: 4.607–20.969, *p* < 0.0001). Diabetic duration, retinopathy, and cataracts were not statistically significantly associated with the risk of at-risk foot conditions.

**Conclusion:**

A high percentage of people with diabetes over 60 years old develop at-risk foot. Fasting blood glucose, income, hypertension, cardiovascular disease, cerebrovascular disease, kidney disease, and peripheral neuropathy are significant independent risk factors for at-risk foot conditions, while diabetic duration, retinopathy, and cataracts are not associated with the risk.

## Highlights

1. *Quantitative risk factor analysis*: This study delineates the specific impacts of fasting blood glucose, income, and comorbidities on the prevalence of high-risk foot conditions in elderly diabetic patients.
2. *Extensive data support*: Utilizing data from 2003 participants, the research provides compelling evidence of the correlations between socioeconomic status, comorbidities, and increased foot risk.
3. *Implications for targeted interventions*: Highlights the importance of addressing modifiable risk factors and comorbid conditions in diabetic foot care strategies.

## 1 Introduction

Diabetic foot disease (DFD) is one of the most severe complications of diabetes mellitus (DM) and the primary cause of diabetes-related hospitalizations and amputations (1). Characterized by high incidence, high disability rate, high mortality, high recurrence, and low healing rates, DFD imposes a significant burden on the healthcare system (2). Both prospective and retrospective studies consistently indicate that loss of protective sensation (LOPS) and peripheral arterial disease (PAD) are associated with an increased risk of diabetic foot ulcers (DFU) (3, 4). Additionally, PAD also increases the risk of ulcer non-healing, infection, and amputation (5).

Early screening to identify at-risk feet is crucial for preventive interventions, which could significantly reduce the incidence of DF and alleviate patient suffering (6). Since the establishment of the International Working Group on the Diabetic Foot (IWGDF) in 1999, increasing emphasis has been placed on at-risk foot, with updates to the risk stratification system in each guideline revision. The IWGDF 2023 risk stratification system includes critical risk factors such as PAD, LOPS, foot deformities, history of foot ulcers, any degree of lower limb amputation, and the end-stage renal disease (7).

Most studies related to at-risk foot are based on databases, cohorts, hospitalized patients, or outpatient studies, lacking comprehensive data on risk factors. According to the latest DFU risk stratification system, there is currently a lack of research or descriptions of the diabetic at-risk foot population. This study aims to conduct a large-scale community screening in the Xiamen city of China, describe the at-risk diabetic foot population in detail according to the latest standards, and analyze the risk factors for at-risk foot.

## 2 Materials and methods

### 2.1 Study population and data sources

This is a population-based cross-sectional study, with the core objective of exploring the prevalence and independent risk factors of diabetic at-risk foot in community-dwelling elderly patients with diabetes mellitus. This study utilized data from the Diabetic Foot Screening Project conducted by the Diabetic Foot Prevention and Treatment Center at the Third Hospital of Xiamen. The data were collected from June 10, 2022, to June 21, 2023, in a cohort established by the center in the Tong’an District of Xiamen for long-term follow-up and intervention studies of the diabetic population, serving as a validation and reliability study for the IWGDF 2023 risk stratification system. The study’s data sources included diabetic foot screening records from the Diabetic Foot Prevention and Treatment Center at the Third Hospital of Xiamen and the community service center’s resident health database.

This study adopted a consecutive recruitment strategy based on community cluster sampling. The sampling frame covered all community-dwelling diabetic patients aged ≥60 years under standardized chronic disease management of family doctors in Tong’an District, Xiamen. During the predefined study period, all eligible patients were notified one by one by their contracted family doctors, and we consecutively enrolled all participants who met the pre-specified inclusion criteria and voluntarily signed the informed consent form for the diabetic foot screening program.

Inclusion Criteria: over 60 years old; clinically confirmed diagnosis of diabetes mellitus. Exclusion Criteria: under 60 years old; Severe systemic disease with a life expectancy of less than 6 months; Temporary residents in the study area; Patients with a confirmed diagnosis of malignant tumor; Patients with incomplete clinical data or invalid questionnaires.

A total of 2,128 eligible elderly diabetic patients were registered in the community chronic disease management system during the study period. Among them, 125 individuals refused to participate in the screening program, with a refusal rate of 5.87%. The primary reasons for refusal were time conflicts and unwillingness to complete the on-site systematic physical examinations. We further compared the age and gender distribution between the refusers and the enrolled participants, and found no statistically significant differences between the two groups (*p* > 0.05), which minimized the potential risk of selection bias in this study. After excluding participants who met the above exclusion criteria, a total of 1,224 eligible patients were finally included in the subsequent statistical analysis, with a complete participant screening flow fully consistent with the pre-specified inclusion and exclusion criteria.

### 2.2 Sample size calculation

The sample size was calculated using the classical formula for cross-sectional prevalence studies. We set a two-sided significance level of *α* = 0.05 (corresponding to Z value of 1.96), a margin of error of 3%, and referred to the 27.8% prevalence of at-risk foot in Chinese diabetic patients reported by domestic peer studies (2). The minimum basic sample size was calculated to be 857 cases. Considering a 15% rate of invalid data and loss to follow-up in community surveys, the final required minimum sample size was adjusted to 986 cases. The 1,224 eligible participants finally included exceeded the pre-calculated minimum sample size, which fully met the statistical requirements of this study and ensured sufficient statistical power for subsequent multivariate regression analysis.

### 2.3 Standard method training

In accordance with the diabetic at-risk foot screening methods provided by the IWGDF 2019 guidelines, all physicians, nurses, technicians, volunteers, and data entry personnel underwent rigorous standardized training in diabetic foot screening prior to the survey. This training included conducting questionnaire, physical examinations, peripheral blood collection, medical waste disposal, and emergency management.

### 2.4 DF risk assessment program

The definition, diagnostic criteria, and 4-level risk stratification of diabetic at-risk foot in this study fully complied with the 2023 International Working Group on the Diabetic Foot (IWGDF) Guidelines on the Prevention and Management of Diabetic Foot Disease, the globally recognized gold standard in this field. All enrolled participants completed standardized screening for three core pathological conditions linked to at-risk foot: loss of protective sensation (LOPS), peripheral arterial disease (PAD), and foot deformities. The screening for foot deformities covered both appearance and structural deformities, including examinations of the skin, nails, arches, toe deformities, and joint mobility, with assessments made by foot and ankle specialists. PAD screening primarily involved palpation of arterial pulses and the Ankle-Brachial Index (ABI), including examinations of the dorsalis pedis and posterior tibial arteries. PAD is considered excluded if the ABI is between 0.9 and 1.3 and arterial pulsation is not reduced. If ABI is less than 0.9 or greater than 1.3, or if arterial pulsation is weakened or absent, PAD is suspected, and further evaluation with a handheld Doppler ultrasound to assess arterial waveforms is conducted. LOPS is identified as abnormalities in pressure or vibratory sensation. This is tested using a 10 g (5.07 Semmes-Weinstein) monofilament to detect pressure/loss of protective sensation and a tuning fork (128 Hz) to assess loss of vibratory sensation, indicative of peripheral neuropathy, the presence of either abnormality confirmed LOPS diagnosis (8). Foot deformities include bony deformities such as hallux valgus, hammertoes, claw toes, mallet toes, and Charcot foot, and nail deformities. Hallux valgus is defined as a deformity of the big toe characterized by lateral deviation and rotation caused by an exostosis related to the inner margin of the metatarsal (bunion) (9). Hammertoe is defined as flexion at the distal and middle interphalangeal joints relative to the proximal phalanges. Claw toe is defined as dorsiflexion at the metatarsophalangeal joint associated with hammertoe (10). Mallet toe is defined by flexion of the distal phalanx relative to the middle phalanx due to contraction of the distal interphalangeal joint (11). Charcot foot is defined as non-infectious destruction of bone and joint tissue, including loss of the arch or rocker-bottom deformity (12). The nail deformities specific to our study refer to ingrown toenails, excluding onychomycosis, pitting, onychoptosis, and onychauxis, which are not mentioned in the guidelines and literature but are included based on clinical experience by the researchers.

Survey data were collected on-site, including basic patient information, family history, history of foot ulcers, and lower limb amputation (minor or severe). The classification criteria for high-risk feet refer to the IWGDF 2023 risk stratification system (Table 1).

**Table 1:** Baseline clinical characteristics of patients in different grades for at-risk foot (*n* = 1,224).

Variable	Total *N* = 1,224	Grade for at-risk foot				*p*
		Very low risk *N* = 814	Low risk *N* = 250	Moderate risk *N* = 130	High risk *N* = 30	
Age	68.86 ± 4.67	68.97 ± 6.03	68.40 ± 5.72	68.74 ± 5.98	70.47 ± 5.70	0.262
Gender						0.740
M	676 (55.23%)	451 (55.41%)	138 (55.20%)	68 (52.31%)	19 (63.33%)	
F	548 (44.77%)	363 (44.59%)	112 (44.80%)	62 (47.69%)	11 (36.67%)	
BMI	24.23 ± 2.81	24.23 ± 3.45	24.09 ± 3.78	24.35 ± 3.22	24.80 ± 3.76	0.714
FBG	12.80 ± 2.81	12.59 ± 4.81	13.25 ± 4.97	12.74 ± 4.51	14.87 ± 5.60	0.026
SBP	147.20 ± 14.70	147.66 ± 18.58	146.66 ± 18.34	145.25 ± 17.68	147.90 ± 19.99	0.532
DBP	77.50 ± 8.02	77.46 ± 9.85	77.74 ± 9.70	77.57 ± 9.87	76.47 ± 9.70	0.918
Course	11.78 ± 5.65	11.55 ± 6.93	12.04 ± 8.83	11.69 ± 8.49	16.10 ± 9.37	0.014
Education						0.457
Undergraduate	23 (1.88%)	13 (1.60%)	6 (2.40%)	4 (3.08%)	0 (0.00%)	
high school education	116 (9.48%)	81 (9.95%)	22 (8.80%)	10 (7.69%)	3 (10.00%)	
junior high school education	223 (18.22%)	155 (19.04%)	45 (18.00%)	16 (12.31%)	7 (23.33%)	
Primary education	385 (31.45%)	242 (29.73%)	79 (31.60%)	53 (40.77%)	11 (36.67%)	
illiteracy	477 (38.97%)	323 (39.68%)	98 (39.20%)	47 (36.15%)	9 (30.00%)	
Occupation						0.166
officer	5 (0.41%)	4 (0.49%)	0 (0.00%)	1 (0.77%)	0 (0.00%)	
Worker	94 (7.68%)	59 (7.25%)	25 (10.00%)	8 (6.15%)	2 (6.670%)	
Farmer	889 (72.63%)	585 (71.87%)	193 (77.20%)	91 (70.00%)	20 (66.67%)	
retiree	236 (19.28%)	166 (20.39%)	32 (12.80%)	30 (23.08%)	8 (26.67%)	
Income*						0.011
less than 300 USD per month	820 (66.99%)	554 (68.06%)	174 (69.60%)	79 (60.77%)	13 (43.33%)	
more than 300 USD per month	404 (33.01%)	260 (31.94%)	76 (30.40%)	51 (39.23%)	17 (56.67%)	
Smoke	232 (18.95%)	149 (18.31%)	56 (22.40%)	23 (17.69%)	4 (13.33%)	0.406
HP	632 (51.63%)	434 (53.32%)	133 (53.20%)	51 (39.23%)	14 (46.67%)	0.024
HD	473 (38.64%)	309 (37.96%)	96 (38.40%)	47 (36.15%)	21 (70.00%)	0.005
CVD	66 (5.39%)	30 (3.69%)	23 (9.20%)	12 (9.23%)	1 (3.33%)	0.001
CBD	27 (2.21%)	16 (1.97%)	6 (2.40%)	0 (0.00%)	5 (16.67%)	<0.001
KD	33 (2.70%)	9 (1.11%)	23 (9.20%)	1 (0.77%)	0 (0.00%)	<0.001
Retinopathy	54 (4.41%)	29 (3.56%)	19 (7.60%)	1 (0.77%)	5 (16.67%)	<0.001
Cataract	156 (12.75%)	96 (11.79%)	49 (19.60%)	10 (7.69%)	1 (3.33%)	<0.001
PN*	63 (5.15%)	12 (1.47%)	36 (14.40%)	10 (7.69%)	5 (16.67%)	<0.001
FH	389 (31.78%)	232 (28.50%)	97 (38.80%)	44 (33.85%)	16 (53.33%)	<0.001
Father	91 (7.43%)	58 (7.13%)	13 (5.20%)	16 (12.31%)	4 (13.33%)	0.047
Mother	103 (8.42%)	79 (9.71%)	22 (8.80%)	2 (1.54%)	0 (0.00%)	0.006
Brother	198 (16.18%)	117 (14.37%)	47 (18.80%)	23 (17.69%)	11 (36.67%)	0.005
Sister	130 (10.62%)	81 (9.95%)	27 (10.80%)	8 (6.15%)	14 (46.67%)	<0.001
Son	34 (2.77%)	14 (1.72%)	17 (6.80%)	3 (2.31%)	0 (0.00%)	<0.001
Daughter	22 (1.80%)	0 (0.00%)	15 (6.00%)	7 (5.38%)	0 (0.00%)	<0.001

All continuous values are presented in mean ± standard deviation. Categorical variables are stated as frequencies and percentages. BMI, body Mass Index; FBG, fasting blood glucos; SBP, systolic blood pressure; DBP, diastolic blood pressure; HP, High pressure; HD, Hyperlipidemia; CVD, cardiovascular disease; CBD, cerebrovascular disease; KD, Kidney disease; FH, family history; PN*, peripheral neuropathy (In this study, peripheral nerve disease was defined as having prior peripheral nerve entrapment or peripheral neuritis, not diabetic peripheral neuropathy). INCOME* the adopted monthly rate of 300 USD is the local minimum subsistence standard.

### 2.5 Statistical analysis

Continuous variables are expressed as mean ± standard deviation, and categorical variables are presented as frequencies (percentages). Smoking status, hypertension, cerebrovascular disease, heart disease, kidney disease, retinopathy, and cataract are considered binary variables. The T-test and Pearson Chi-square/Fisher’s exact test are used to compare quantitative and qualitative variables between groups, respectively. Two sets of logistic regression models were constructed to explore factors associated with diabetic at-risk foot. First, an ordinal logistic regression model was applied, with the 4-level IWGDF risk grade (very low, low, moderate, high risk) as the response variable, to identify independent risk factors for at-risk foot. A two-step variable selection strategy was adopted: variables with *p* < 0.05 in univariate analysis, combined with clinical relevance as established risk factors in existing literature, were included in the multivariable model. Age and gender were forced into the model as conventional demographic confounders regardless of their univariate significance. Key confounding factors, including diabetes duration, body mass index (BMI), smoking status, peripheral neuropathy history, and fasting blood glucose (FBG, used as the glycemic control indicator for this community-based screening study, given the unavailability of recent HbA1c results in some participants), were fully adjusted for in the model. Second, a binary logistic regression model was used for supplementary analysis, with at-risk foot (vs. very low risk) as the outcome, to verify the robustness of the findings.

Statistical analyses are performed using Empowers tats (version 4.2) and R.A two-sided *p*-value <0.05 was defined as statistically significant.

## 3 Results

### 3.1 Demographic characteristics

**Figure 1:**
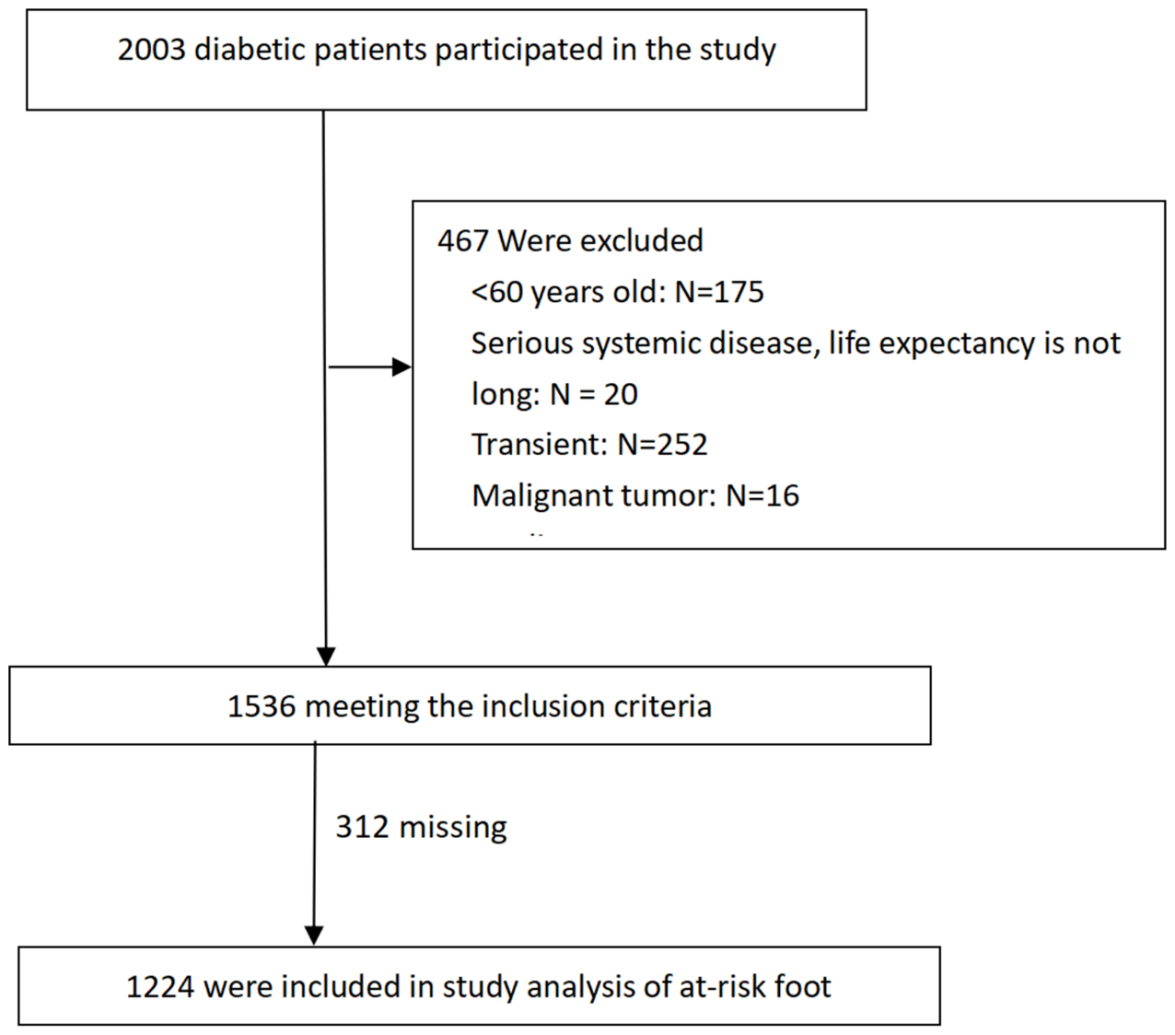
Flow chart of data sample generation for inclusion in the primary analysis.

**Figure 2:**
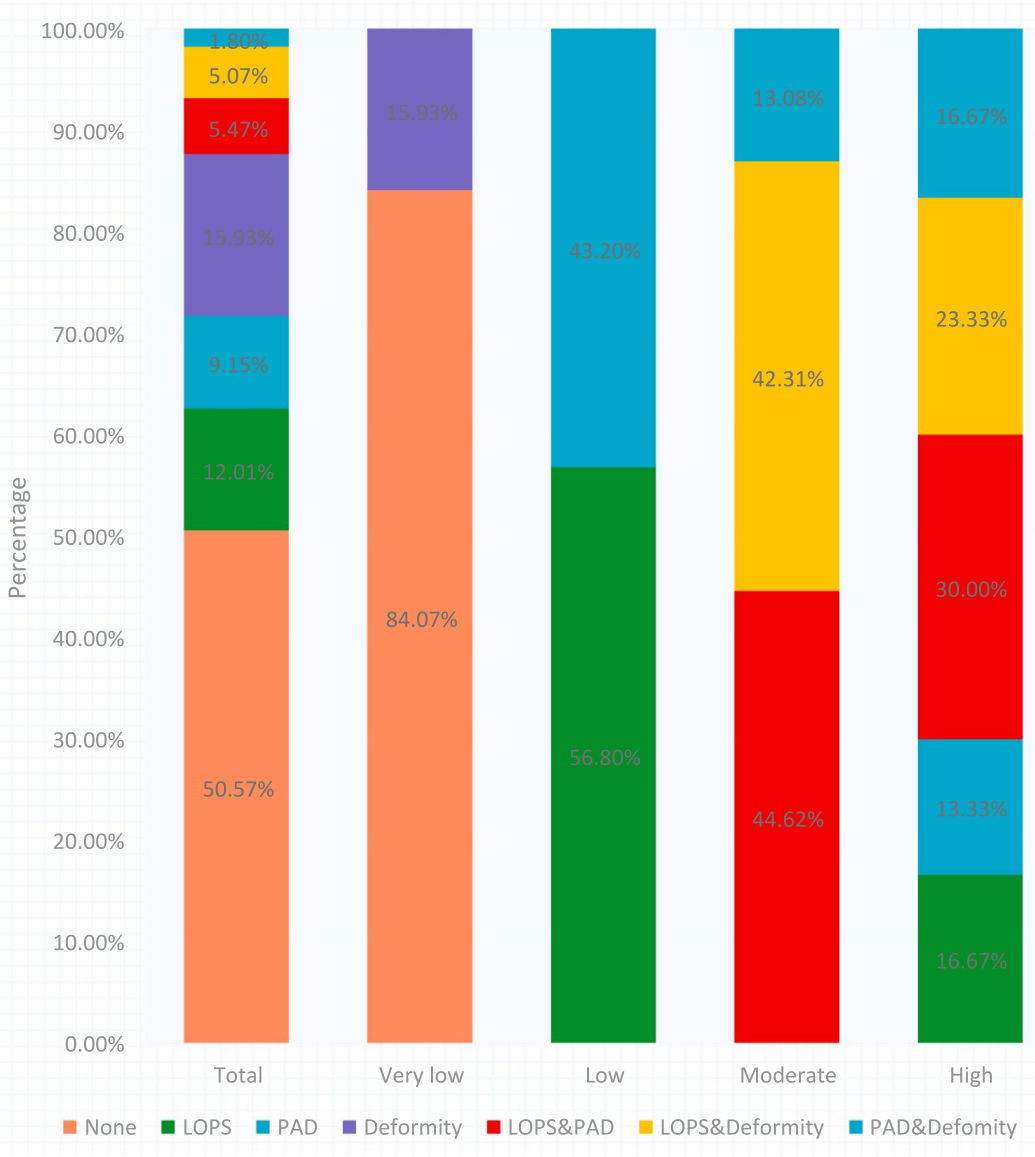
The proportion of PAD, LOPS, and foot deformity in different grades for at-risk foot. The percentage with different colors indicates the proportion in different grading groups.

**Figure 3:**
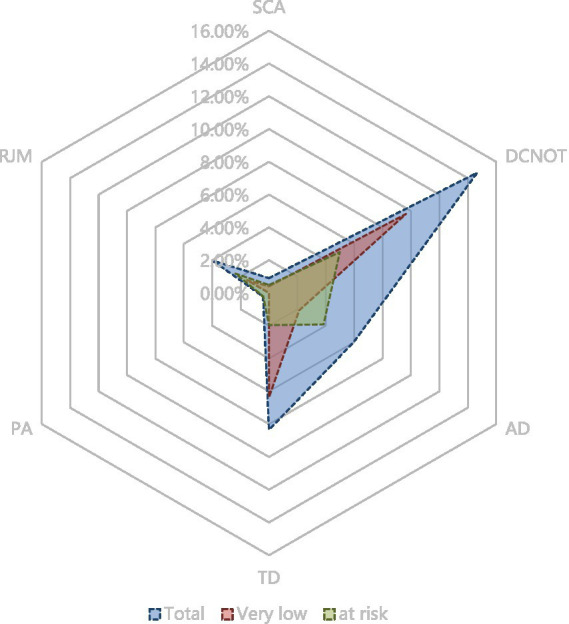
The radar chart shows the proportion and distribution of different types of foot deformities in different grading groups with different colors. SCA, Severe callus accumulation; DCNOT, Damaged curly nails or toenails; AD, Arch deformity; TD, Toe deformity; PA, Partial amputation; RJM, Restriction of joint movement.

The study enrolled 2003 diabetic patients, with an average age of 68.90 ± 4.67 years, of which 99.1% had Type 2 diabetes. Based on the inclusion criteria and missing records, 1,224 participants were ultimately included in the analysis for at-risk diabetic foot (Figure 1). Among these, 55.2% (676) were male and 44.7% (548) were female. Those with an education level above high school accounted for 11.36% (139), farmers comprised 72.63% (889), and individuals with a monthly income below 300 USD represented 66.99% (820). The prevalence of smoking history was 18.87% (231), history of hypertension 51.63% (632), hyperlipidemia 38.64% (473), cardiovascular diseases 5.39% (66), non-cerebrovascular diseases 2.21% (27), kidney diseases 2.70% (33), awareness of retinopathy 4.41% (54), and cataracts 12.75% (156). A history of peripheral neuropathy was reported by 5.15% (63). A family history of diabetes was noted in 31.78% (389), with fathers accounting for 7.43% (91), mothers 8.42% (103), brothers 16.18% (198), sisters10.62% (130), sons 2.77% (34), and daughters1.80% (22). The prevalence of at-risk feet among all participants was 33.50% (410). The low-risk group comprised 60.98% (250), followed by the moderate-risk group at 31.71% (130), and the high-risk group at 7.32% (30). Figure 2 displays the prevalence of PAD, LOPS, or deformities at different risk levels during screening. LOPS was present in 22.54% (276) of participants, PAD in 16.42% (201), and foot deformities in 22.79% (279). It was also found that 5.47% (67) had both PAD and LOPS, 5.07% (62) had both LOPS and foot deformities, and 1.80% (22) had both PAD and foot deformities. Figure 3 shows the distribution of foot deformity types across the entire risk group and the very low-risk group. According to the IWGDF 2023 classification standards, foot deformities in the very low-risk group accounted for 23.90% (195), in the moderate-risk group 55.3% (72), and in the high-risk group 40.00% (12). No foot deformities were found in the low-risk group. Among the entire population, severe callus accumulation was 0.90% (11), nail deformity was 14.62% (179), arch deformities 5.96% (73), toe deformities 8.33% (102), history of ulcers 2.04% (25), minor amputations 0.41% (5), with no cases of above-ankle amputations, and 3.9% (48) had limited mobility of the metatarsophalangeal or ankle joints. Additionally, 2.6% of the population wore inappropriate footwear.

### 3.2 Analysis of population characteristics in at-risk diabetic foot

This study aimed to evaluate the baseline clinical characteristics of 1,224 diabetic patients categorized into four risk categories for foot-related complications: very low risk, low risk, moderate risk, and high risk. The analysis focused on age, gender, body mass index (BMI), fasting blood glucose (FBG), and other relevant clinical parameters. The average FBG level was highest in the at-risk group (14.87 ± 5.60 mg/dL), with this variation being statistically significant (*p* = 0.026), suggesting a potential correlation between elevated fasting blood glucose levels and increased foot risk. Differences in education, occupation, and income levels were observed across groups, with significant discrepancies in income (*p* = 0.011); Compared with the monthly personal disposable income >300 USD (Local minimum subsistence standard for individuals) group, the monthly personal disposable income <300 USD group, a higher proportion of patients in the high-risk group had a monthly income exceeding 300 USD. Other clinical parameters such as systolic blood pressure (SBP), diastolic blood pressure (DBP), and disease duration showed no significant differences between the risk groups, except for a notably longer disease duration in the high-risk group (16.10 ± 9.37 years, *p* = 0.014). Regarding complications, the prevalence of hypertension (HP), hyperlipidemia (HD), cardiovascular diseases (CVD), cerebrovascular diseases (CBD), kidney diseases (KD), retinopathy, cataracts, peripheral neuropathy (PN), and family history showed significant differences. Notably, the prevalence of KD, retinopathy, cataracts, and PN was significantly higher in the high-risk group (*p* < 0.001) (Table 1).

### 3.3 Risk factors for at-risk diabetic foot

In our study, we utilized a multivariate logistic regression model to assess and identify independent risk factors affecting foot risk status. After adjusting for potential confounders, we found that fasting blood glucose (FBG), income, hypertension (HP), cardiovascular diseases (CVD), cerebrovascular diseases (CBD), kidney diseases (KD), and peripheral neuropathy (PN) were significant independent risk factors for foot risk status. Specifically, our analysis indicated that for each increase of 1 mmol/L in FBG, the probability of foot risk status increased by 4.8% (OR = 1.048, 95% CI: 1.018–1.078, *p* = 0.001). Additionally, for each increase of 1 USD in income, the probability of foot risk status increased by 37.4% (OR = 1.374, 95% CI: 1.022–1.848, *p* = 0.035). In contrast, the presence of hypertension reduced the probability of foot risk status by 37.4% (OR = 0.626, 95% CI: 0.464–0.845, *p* = 0.002). Cardiovascular diseases significantly increased the probability of at-risk foot status by approximately 90.6% (OR = 1.906, 95% CI: 1.091–3.328, *p* = 0.023). Cerebrovascular diseases significantly reduced the probability of at-risk foot status (OR = 0.226, 95% CI: 0.064–0.803, *p* = 0.022). Kidney diseases significantly increased the probability of at-risk foot status by approximately 257.8% (OR = 3.578, 95% CI: 1.567–8.171, *p* = 0.025). Most notably, peripheral neuropathy significantly increased the probability of at-risk foot status by approximately 882.9% (OR = 9.829, 95% CI: 4.607–20.969, *p* < 0.0001). However, the duration of diabetes, retinopathy, and cataracts were not associated with the risk of diabetic at-risk foot, showing no statistical significance (Table 2).

**Table 2:** Multivariate analysis results of risk factors of at-risk foot.

Variable	Coefficient	SE	OR	95% CI	*p*
FBG	0.029	0.013	1.048	1.018 1.078	0.001
course	0.005	0.008	1.012	0.994 1.030	0.196
Income	0.106	0.135	1.374	1.022 1.848	0.035
HP	0.168	0.133	0.626	0.464 0.845	0.002
HD	0.024	0.137	1.337	0.985 1.815	0.063
CVD	0.742	0.270	1.906	1.091 3.328	0.023
CBD	0.257	0.479	0.226	0.064 0.803	0.022
KD	1.042	0.436	3.578	1.567 8.171	0.025
Retinopathy	0.588	0.417	1.228	0.542 2.780	0.280
Cataract	0.149	0.207	0.858	0.538 1.370	0.522
PN	2.420	0.430	9.829	4.607 20.969	<0.000
FH	0.348	0.137	1.341	0.988 1.820	0.059

The multiple logistic regression model was utilized to evaluate the independent risk factors for four grades of the at-risk foot: very low, low, moderate and high risk; FBG, fasting blood glucos. HP, High pressure; HD, Hyperlipidemia; CVD, cardiovascular disease; CBD, cerebrovascular disease; KD, Kidney disease; PN, peripheral neuropathy; FH, family history.

## 4 Discussion

Diabetic foot is one of the most common, costly, and severe complications of diabetes, with an estimated limb or part of a limb lost every thirty seconds. Consequently, diabetic foot (DF) represents a significant public, economic, and social health challenge (13). However, not all individuals with diabetes are at risk for ulceration. The primary risk factors include loss of protective sensation (LOPS), peripheral arterial disease (PAD), and foot deformities (7). LOPS diminishes the foot’s response to harmful external stimuli, often leading to ulcers due to unperceived repetitive pressure in specific areas; PAD is considered an independent risk factor that impacts wound healing and the necessity for amputation (14–16). Most research focuses on the incidence of LOPS and PAD, with less attention given to foot deformities. Diabetic peripheral neuropathy (DPN), PAD, and foot deformities are relatively common among diabetic patients, inflicting physical and emotional distress on patients and their families, thereby affecting their quality of life (17). Understanding the prevalence and risk factors of DPN, PAD, and foot deformities is crucial for developing preventive strategies for diabetic foot in diabetic patients. Therefore, we conducted a multi-community diabetic foot screening across nine communities in Xiamen, ultimately analyzing 1,224 cases from the natural diabetic population to determine the prevalence and risk factors of diabetic foot disease among individuals over 60 years old.

This study found that the incidence of at-risk diabetic foot in the diabetic population over 60 years old was 33.50%, which differs from the 27.8% reported by Ren et al. (2), possibly due to differences in the study populations and regions. Chen et al. (18), who included 13,315 diabetic patients in their study, reported that 4.9% of the patients had PAD and 43.9% had DPN. This contrasts significantly with our findings, where 16.42% of participants had PAD and 22.54% had LOPS. The discrepancy may be due to Chen et al. using only a 10 g monofilament for diagnosing DPN and an ankle-brachial index (ABI) less than 0.9 for diagnosing PAD, without considering decreased vibratory perception and ABI greater than 1.3, and only reporting the incidence of diabetic peripheral neuropathy and PAD without attention to foot deformities. Liu et al. (17) conducted an epidemiological survey of diabetic patients in outpatient clinics across 11 hospitals in Beijing, focusing on foot deformities and reported a deformity incidence of 29.7%, similar to our finding of 22.79%. Our study, based on the IWGDF 2023 stratification, not only reports the incidence of LOPS, PAD, and foot deformities but also addresses the incidence of conditions combining LOPS with PAD, LOPS with foot deformities, and PAD with foot deformities. We also found that even in the very low-risk group, the incidence of foot deformities was as high as 23.90%, a clinically significant issue since even non-diabetic patients with foot deformities can develop skin ulcers at the deformity site. Therefore, whether interventions for foot deformities in the very low-risk group are warranted merits further study. The IWGDF guidelines focus more on the contribution of LOPS and PAD to the risk of diabetic foot and also consider foot deformities but do not describe toenail deformities. Our study addresses toenail deformities and inappropriate footwear, as we have observed in clinical practice that some cases requiring toe amputation in diabetic foot are due to toenail deformities and improper toenail trimming. Therefore, this aspect was included in our screening process and presented in the results. Additionally, we focused on the mobility of the foot and ankle joints, as joint mobility is crucial in the gait cycle, and limited mobility can lead to abnormal pressure in certain areas of the footed (19–21), similar to foot deformities, becoming a risk factor for diabetic foot. Due to the challenges of community screening, the typical study populations in Chinese research are hospitalized or outpatient diabetic patients, which do not represent the prevalence and characteristics of the natural diabetic population. Based on the latest guidelines and comprehensive screening, our study more objectively describes the prevalence and characteristics of the natural diabetic population over 60 years old in China.

Our study results show that each increase of 1 mmol/L in fasting blood glucose levels raises the probability of a high-risk foot condition by 4.8%. This finding is consistent with prior research indicating that poor glycemic control is a significant risk factor for diabetic foot (2, 22). However, other studies have noted that good glycemic control does not necessarily reduce the incidence of PAD (23). Additionally, we found that an increase in income level significantly correlates with an increased risk of foot complications. This may suggest that individuals with higher economic status may have more opportunities to engage in activities that increase the risk of foot complications, or it could reflect other unadjusted confounding factors. This was only a cross-sectional study, and the observed associations need to be confirmed by further research.

It is noteworthy that although hypertension is commonly considered a risk factor for cardiovascular diseases, in our study, we found that the prevalence of diabetic at-risk foot was lower in the hypertensive population, which may be explained by the following reasons: (1) Treatment and health management effects: Diabetic patients with hypertension received more frequent community follow-up, more standardized antihypertensive and hypoglycemic treatment, and better diabetes management adherence, which indirectly reduced at-risk foot risk, rather than hypertension itself having a protective effect; (2) Residual confounding: Unmeasured factors such as foot care awareness and self-management behavior may still exist despite multivariable adjustment; (3) Survivor bias: The study population was elderly patients aged ≥60 years, and hypertensive patients who survived and participated in the screening may have better overall health status and treatment adherence. Conversely, cardiovascular diseases and kidney diseases significantly increased the probability of high-risk foot conditions, consistent with previous studies indicating that these systemic diseases are associated with an increased risk of diabetic foot (24–26).

Most notably, the prevalence of diabetic at-risk foot in the population with peripheral neuropathy was 7.8 times that in the population without peripheral neuropathy. This finding underscores the importance of closely monitoring individuals with a history of conditions such as peripheral nerve compression or peripheral neuritis, as they play a significant role in the risk of diabetic foot. Numerous studies have been conducted on the relationship between Carpal Tunnel Syndrome (CTS) and diabetic peripheral neuropathy (DPN), showing consistent conclusions that DPN patients have an increased risk of developing CTS compared to diabetic patients without DPN (27–30), aligning with our findings. This highlights the indispensable role of understanding the history of peripheral neuropathic diseases in the foot risk assessment of diabetic patients.

It is noteworthy that although our results did not show a direct association between the duration of diabetes, retinopathy, and cataracts with an increased risk of diabetic foot, this does not imply that these factors are unimportant in diabetes management. Some studies have found that the occurrence of diabetic foot is related to factors such as the duration of diabetes, retinopathy, and cataracts (17, 31, 32), which may indirectly affect foot health through other mechanisms.

In summary, our study provides an in-depth understanding of the risk factors for diabetic foot and emphasizes the importance of comprehensive management strategies, including glycemic control, management of cardiovascular and kidney diseases, and early diagnosis and treatment of neuropathy. Future research should further explore how these risk factors affect foot health through specific mechanisms and how effective prevention strategies can reduce the incidence of diabetic foot.

Our study has several strengths and unique features, including multiple community health service centers, a broad sample population data, and a standardized team of examiners trained in diabetic foot screening, which ensures a high degree of homogeneity and representativeness in the results. However, our study has certain limitations. Firstly, our sample is limited to Xiamen, China, and the conclusions may not be generalizable to other regions. Secondly, our analysis population consists of diabetic individuals over 60 years old, and the conclusions may not be applicable to those under 60 years old. We emphasizing that the cross-sectional design cannot determine the temporal sequence between exposure and outcome, thus cannot support causal inference.

## Data Availability

The raw data supporting the conclusions of this article will be made available by the authors, without undue reservation.
